# Uncertainty Quantification in Medical Image Segmentation: A Comprehensive Survey

**DOI:** 10.3390/jimaging12080341

**Published:** 2026-07-28

**Authors:** Seyed Sina Ziaee, Katie Ovens

**Affiliations:** Department of Computer Science, University of Calgary, 2500 University Dr NW, Calgary, AB T2N 1N4, Canada

**Keywords:** uncertainty quantification, medical image segmentation, Bayesian methods, Monte Carlo dropout, deep ensembles, deterministic uncertainty methods, test-time data augmentation, hybrid models, aleatoric uncertainty, epistemic uncertainty

## Abstract

Uncertainty quantification (UQ) in medical image segmentation is essential for ensuring the reliability and interpretability of deep learning models in clinical decision-making. While convolutional neural networks (CNNs) and transformer-based architectures have achieved remarkable segmentation performance, they often provide deterministic outputs without accounting for uncertainty, which can lead to overconfident predictions in ambiguous cases. This paper presents a comprehensive survey of UQ techniques in medical image segmentation, categorizing existing approaches into Bayesian methods, deep ensembles, deterministic methods, test-time data augmentation, and hybrid models, while treating foundation-model-based UQ as a separate cross-cutting category. We examine key methodologies, including Monte Carlo dropout, Bayesian neural networks, variational inference, and ensemble learning, discussing their advantages and limitations in addressing aleatoric and epistemic uncertainties. Additionally, we explore the clinical relevance of UQ by reviewing its applications in brain tumor segmentation, cardiac imaging, lung nodule detection, and other medical domains. The paper also highlights key evaluation metrics, such as calibration errors, uncertainty–error correlation, and visual interpretability, to assess the effectiveness of UQ methods. Finally, we discuss challenges and future research directions, emphasizing the need for scalable, interpretable, and clinically actionable uncertainty quantification strategies to improve trust in AI-assisted medical image analysis.

## 1. Introduction

Uncertainty quantification (UQ) has become a key component of measuring the trustworthiness of artificial intelligence (AI), particularly in high-stakes domains where erroneous predictions carry significant consequences. UQ provides probabilistic estimates of model confidence, enabling users to distinguish reliable predictions from uncertain ones and fostering informed decision-making. This capability is important in the healthcare domain, where AI tools must balance precision with transparency to gain clinical acceptance.

Medical imaging—encompassing modalities such as MRI, CT, and ultrasound—plays an important role in modern medicine, aiding in diagnosis, treatment planning, and disease monitoring. AI has revolutionized the analysis of medical images through tasks like classification (e.g., identifying pathology presence/absence) [1], detection (e.g., localizing lesions) [2], and segmentation (e.g., delineating anatomical structures pixel-wise) [3]. Among these applications, medical image segmentation is uniquely challenging due to its fine-grained requirements: it demands precise localization of boundaries in inherently noisy, low-contrast, or artifact-prone imaging data. Variability in anatomy, imaging protocols, and pathological presentations further complicates this task and amplifies the need for interpretable AI solutions.

**Figure 1:**
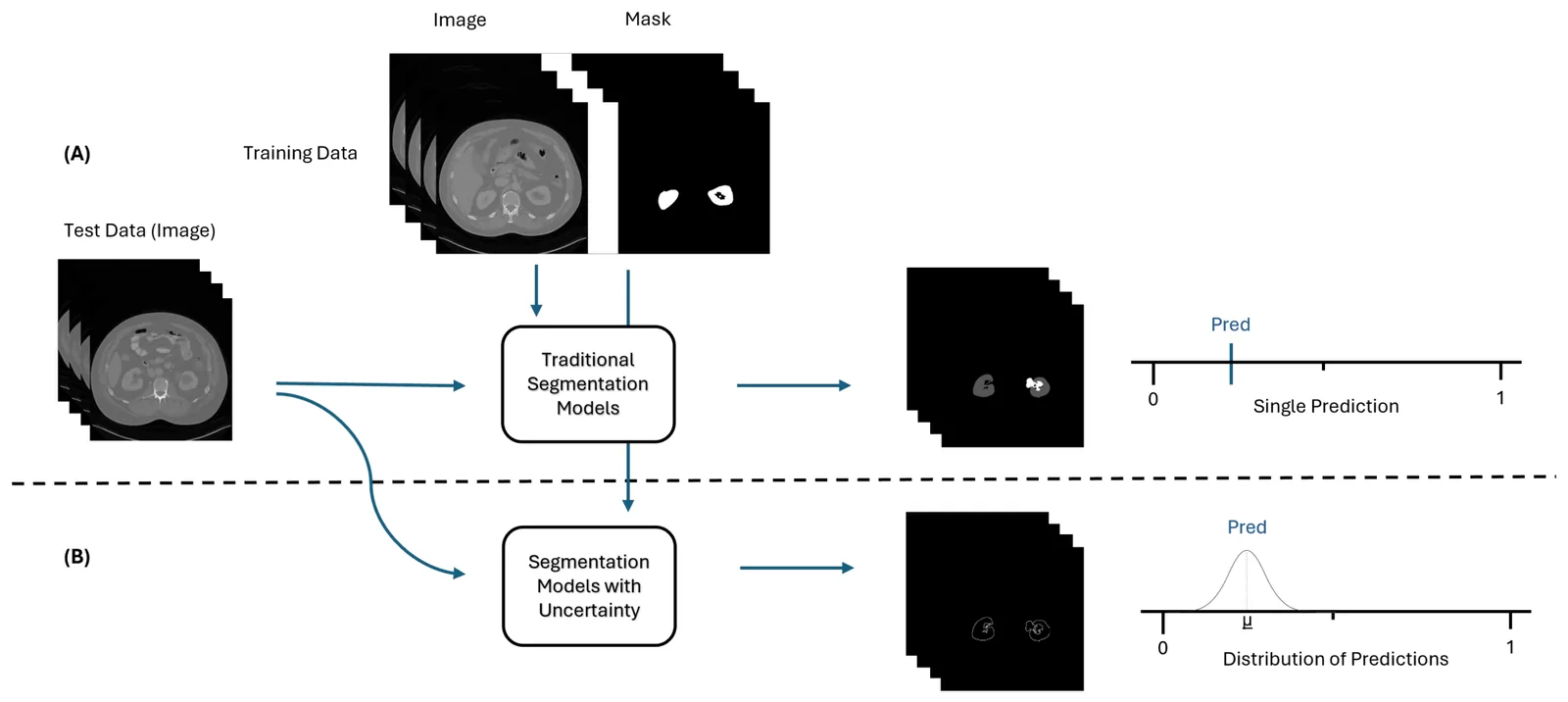
Comparison of traditional segmentation models and uncertainty-aware segmentation models. In traditional segmentation models (**A**), a single prediction is made for each pixel. In contrast, uncertainty-aware models (**B**) generate a distribution of predictions for each pixel.

While deep learning models, including convolutional neural networks (CNNs) and transformer-based architectures [4,5], have achieved state-of-the-art segmentation performance, their deterministic outputs often mask critical uncertainties. A prominent example in medical image segmentation is the delineation of kidneys, tumors, and cysts using U-Net [4], a foundational architecture widely adopted for its effectiveness across diverse imaging modalities. U-Net has been adapted across applications in 2D, 2.5D, and 3D configurations, demonstrating versatility in handling spatial complexity (Figure 1A). However, traditional U-Net implementations produce deterministic pixel-wise predictions, which lack insight into the confidence of the model. Incorporating uncertainty quantification transforms the output of the model from a single segmentation mask into a probabilistic distribution for each pixel (Figure 1B). This shift enables clinicians to assess prediction reliability, particularly in ambiguous regions such as poorly defined tumor boundaries or artifact-affected areas. Recent advancements extend beyond U-Net’s architecture, exploring hybrid models (e.g., transformer-enhanced networks) and novel UQ strategies. These include integrating uncertainty estimation directly into segmentation pipelines—via Bayesian layers [6], Monte Carlo dropout [7], or ensemble methods [8]—or using post hoc uncertainty evaluation to flag low-confidence predictions. Such approaches aim to balance segmentation accuracy with interpretability, which is required for clinical deployment.

Key challenges persist in translating UQ from theory to clinical practice. Interpretability remains a hurdle as uncertainty maps must be intuitive for non-technical users, and their integration into workflows requires careful validation. In addition, more general models that are usable in multimodal images are required. Moreover, the computational cost of UQ methods and the lack of standardized evaluation metrics complicate comparisons across studies.

In this work, we provide a comprehensive review of uncertainty quantification in medical image segmentation. We include an overview of the sources of uncertainty, then critically analyze UQ methodologies, evaluation metrics, and applications, drawing insights from the recent literature. We also explore open challenges in applying these methods in clinical settings. This review aims to encourage the development of reliable, uncertainty-aware segmentation tools that improve patient care by connecting technical advances with clinical needs.

## 2. Survey Methodology

This study was conducted as a structured narrative review rather than a formal systematic review. Literature was identified through searches of Google Scholar and Scopus using combinations of the terms “uncertainty estimation,” “uncertainty quantification,” and “medical image segmentation.” The review focused primarily on studies published from 2021 to February 2025, while earlier foundational studies were included when necessary to explain the development of major uncertainty quantification methods.

Studies were considered relevant when they focused on uncertainty quantification in medical image segmentation, introduced or evaluated an uncertainty estimation approach, or provided findings relevant to calibration, uncertainty–error correspondence, clinical applicability, or probabilistic segmentation. Studies focusing exclusively on non-medical images, classification or detection without a segmentation component, or methods without an uncertainty-related analysis were excluded from the main synthesis.

The selected literature was organized according to the uncertainty quantification strategy, including Bayesian methods, deep ensembles, deterministic methods, test-time data augmentation, hybrid approaches, and foundation-model-based approaches. Information regarding the employed method, dataset, imaging modality, application area, and uncertainty estimation strategy was extracted and synthesized.

Because the review was not originally designed as a formal systematic review, records of database-specific retrieval counts, duplicate removal, sequential screening decisions, and study-level quality assessments were not maintained. Consequently, a retrospective PRISMA flow diagram could not be produced without introducing potentially unreliable estimates. This limitation is acknowledged, and future updates of this survey should use a prospectively registered search protocol and standardized quality-assessment procedure.

## 3. Types of Uncertainty

**Figure 2:**
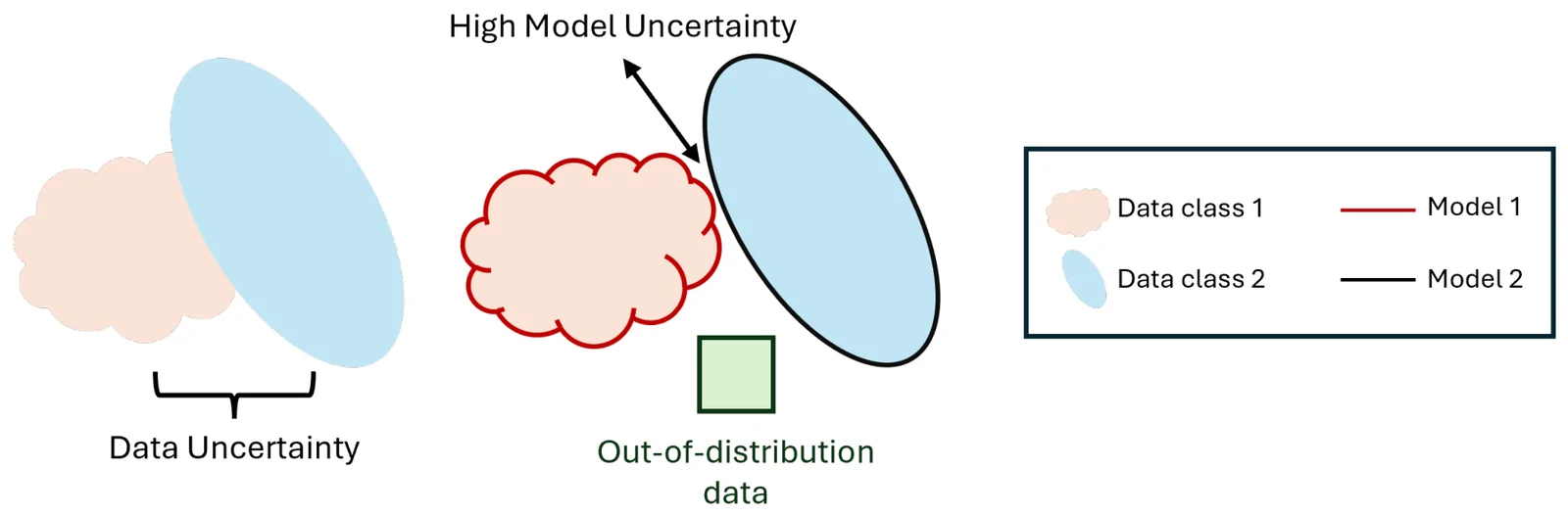
Aleatoric (data) uncertainty in comparison with epistemic (model) uncertainty in segmentation tasks. On the left, data uncertainty is depicted as overlapping regions of two data classes, where inherent noise or ambiguity in the data themselves makes classification uncertain. On the right, model uncertainty is shown with a model struggling to classify data points, especially for out-of-distribution data (green square), leading to high epistemic uncertainty. The red and black outlines represent two different model predictions, emphasizing how models can struggle with unfamiliar or ambiguous inputs.

In the context of deep learning, uncertainty refers to the lack of confidence a model has in its predictions. It is a measure of model ambiguity regarding the correct output for a given input. As shown in Table 1 and Figure 2, there are two main types of uncertainty to quantify in deep learning models. (See Table 1 for more in-depth comparison).

### 3.1. Aleatoric Uncertainty

Also referred to as data uncertainty, aleatoric uncertainty arises due to inherent noise or randomness in the data. It can arise from low resolution or poor image quality, noise in data, or ambiguous annotation. This type of uncertainty cannot be reduced by collecting more data, but it can be estimated by training the model to output a distribution over possible predictions, rather than a single-point estimation.

### 3.2. Epistemic Uncertainty

Also referred to as model uncertainty, epistemic uncertainty results from a lack of knowledge or limitations in the model and training data. It is reducible with better data or improved models. Some of the major sources of this type of uncertainty are insufficient or biased training data, incomplete annotations, inter-observer variability in annotations, and poor generalization to unseen domains.

## 4. Methods of Uncertainty Quantification

**Figure 3:**
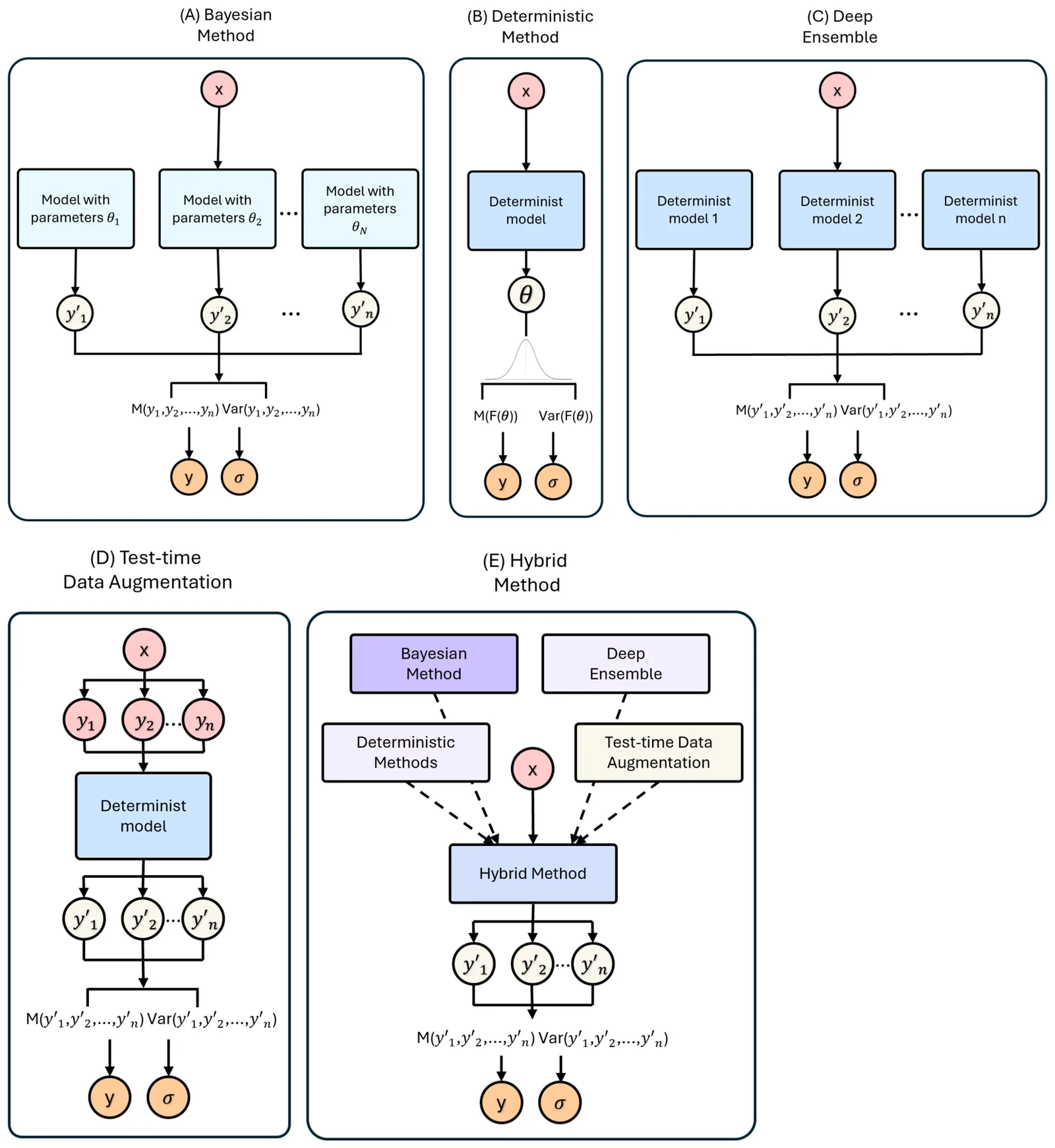
Methods of uncertainty quantification in medical image segmentation. Bayesian methods (**A**) generate multiple predictions by sampling model parameters or latent variables. Deterministic methods (**B**) estimate uncertainty directly from model outputs or learned uncertainty representations. Deep ensembles (**C**) combine predictions from independently trained models. Test-time data augmentation (**D**) measures prediction variability across transformed versions of the input. Hybrid methods (**E**) combine two or more uncertainty quantification strategies. For methods producing multiple predictions, predictive uncertainty can be summarized using statistics such as the predictive mean and variance.

Five major mechanism-based families are commonly used for quantifying uncertainty in medical image segmentation: Bayesian methods, deep ensembles, deterministic methods, test-time data augmentation, and hybrid methods. In addition, foundation-model-based uncertainty quantification is considered as a separate cross-cutting category. This category is defined by the use of large pretrained and promptable segmentation models rather than by a single uncertainty estimation mechanism, because foundation-model-based approaches may employ deterministic, Bayesian, ensemble, augmentation-based, or hybrid strategies. Figure 3 illustrates their general workflows, while Table 2 provides a comparative analysis of their underlying principles, advantages, limitations, and typical application scenarios.

For uncertainty quantification methods that generate *S* predictions ${\left\{y^{\left(s\right)}\right\}}_{s=1}^{S}$, the pixel- or voxel-wise predictive mean and variance can be estimated as:

(1) $$\hat{y}=\frac{1}{S}\sum_{s=1}^{S}y^{\left(s\right)},$$

(2) $$\sigma^{2}=\frac{1}{S}\sum_{s=1}^{S}{\left(y^{\left(s\right)}\right)}^{2}-{\hat{y}}^{\;2}.$$

Here, *S* is the number of predictions, *s* indexes an individual prediction, $y^{\left(s\right)}$ is the *s*th pixel- or voxel-wise prediction, $\hat{y}$ is the predictive mean, and $\sigma^{2}$ is the predictive variance. Depending on the uncertainty quantification method, these predictions may be generated by stochastic forward passes, independently trained ensemble members, or transformed versions of the input.

The selection of an uncertainty quantification method depends on the required balance among uncertainty quality, computational cost, implementation complexity, and clinical applicability. Bayesian and ensemble-based methods generally provide more comprehensive estimates of epistemic uncertainty but require repeated inference or the training of multiple models. Deterministic methods provide faster alternatives that are more suitable for real-time deployment, although they may not fully capture uncertainty caused by limited model knowledge. Test-time data augmentation is model-agnostic and can be applied to pretrained systems, whereas hybrid methods combine complementary strategies at the cost of increased computational and implementation complexity. These differences are summarized in Table 2.

Foundation-model-based methods are presented separately because their defining characteristic is the use of a large pretrained and promptable architecture. They remain cross-cutting with respect to the underlying uncertainty mechanism and may incorporate deterministic, Bayesian, augmentation-based, ensemble, or hybrid uncertainty estimation.

While Table 2 summarizes the methodological principles and application scenarios of the reviewed approaches, Table 3 provides a unified cross-method comparison based on uncertainty estimation quality, computational cost, calibration performance, scalability, interpretability, and clinical applicability. These dimensions are particularly important when selecting an uncertainty quantification strategy for practical medical image segmentation systems.

The assessments in Table 3 are qualitative and summarize commonly reported characteristics rather than universal rankings. Actual performance depends on the segmentation architecture, dataset, imaging modality, uncertainty definition, calibration procedure, and evaluation protocol.

Overall, no uncertainty quantification method is optimal across all medical image segmentation settings. Bayesian methods provide a principled probabilistic framework but may be difficult to scale to large 3D models. Deep ensembles commonly provide strong empirical uncertainty estimates and calibration, although their training, storage, and inference requirements may limit clinical deployment. Monte Carlo sampling and test-time augmentation offer practical alternatives when repeated inference is acceptable, whereas deterministic approaches are better suited to real-time or resource-constrained workflows but may incompletely capture epistemic uncertainty. Hybrid methods can combine complementary uncertainty sources, but their computational cost, calibration complexity, and limited interpretability remain important barriers.

Therefore, method selection should be guided by the intended clinical action. Applications requiring rapid quality control may favor deterministic or lightweight single-model approaches, while safety-critical offline workflows may justify Bayesian, ensemble, or hybrid methods. Future work should focus on efficient methods that preserve calibration and robustness under domain shift while reducing the computational and memory requirements associated with repeated inference and multiple models.

### 4.1. Bayesian Methods

Bayesian methods incorporate probability distributions over model parameters or outputs to capture uncertainty. They often involve Bayesian inference techniques such as variational inference, Monte Carlo sampling, or explicit probabilistic modeling. They provide a principled approach to model uncertainty, capturing both epistemic and aleatoric uncertainties. Bayesian uncertainty estimation incorporates a probability distribution over model parameters θ or outputs *y*. Given an input *x*, the Bayesian posterior over predictions is computed as:

(3) p(y|x,D)=∫p(y|x,θ)p(θ|D)dθ.

Since the true posterior distribution p(θ∣D) is generally intractable, variational inference approximates it using a tractable variational distribution q(θ). The evidence lower bound (ELBO) is defined as:

(4) $$L_{\mathrm{VI}}=E_{q(\theta )}\left[\log p(D|\theta )\right]-D_{\mathrm{KL}}\left(q(\theta )\;∥\;p(\theta )\right),$$

where the Kullback–Leibler divergence is given by:

(5) $$D_{\mathrm{KL}}\left(q(\theta )\;∥\;p(\theta )\right)=\int q\left(\theta \right)\log\left(\frac{q(\theta )}{p(\theta )}\right)d\theta .$$

Here, $L_{\mathrm{VI}}$ is maximized to obtain a variational distribution q(θ) that approximates the true posterior p(θ∣D). The distribution q(θ) denotes the variational approximation, while p(θ) denotes the prior distribution over the model parameters.

For Monte Carlo dropout, predictive uncertainty is estimated by keeping dropout active during inference and generating *S* stochastic predictions, where $y^{\left(s\right)}=f_{\theta_{s}}\left(x\right)$ and $\theta_{s}$ represents the effective model parameters induced by the *s*th dropout mask. The predictive mean and variance are computed using Equations (1) and (2). Greater predictive variance indicates greater model uncertainty.

The main categories of Bayesian uncertainty quantification methods are discussed in the following subsections.

#### 4.1.1. Variational Inference and Probabilistic Models

Variational inference and other probabilistic models address uncertainty quantification by placing distributions over latent variables (or over model parameters) and then approximating these distributions during training. Examples of these types of methods include variational autoencoders to capture fine-grained uncertainty at the pixel level [9], and Variational Moment Propagation (VMP) extended this idea by propagating variational distributions hierarchically, which allows for more reliable uncertainty estimation in complex datasets [10].

Another common approach for uncertainty modeling utilizes Bayesian neural networks, which provide a framework for incorporating uncertainty directly into the parameters of a model. Jones et al. made use of Dirichlet priors in a direct computation setup [11]. Zhao et al. focused on efficient Bayesian inference for nnU-Net [12], where they proposed a scheme for the posterior sampling of weight space for Bayesian uncertainty estimation [13]. Further, Ziaee et al. focused on utilizing Stochastic Gradient Descent with Warm Restarts (SGDR), where the model is trained to escape local minima, creating multiple checkpoints at key training stages, then used to generate an ensemble of predictions, leading to produce uncertainty maps to enhance segmentation reliability [14].

One of the breakthroughs toward uncertainty estimation in deep learning was from Kohl et al., who introduced the Probabilistic U-Net, a seminal work that incorporated uncertainty directly into the segmentation task by modifying the standard U-Net with probabilistic layers. This allowed the model to estimate the uncertainty in pixel-wise predictions [15]. On a similar note, Baumgartner et al. propose PHiSeg, a method that applies hierarchical probabilistic modeling to medical image segmentation. This approach models uncertainty at multiple scales, capturing both global and local variations in the data [3].

In addition, various studies have independently addressed the challenge of quantifying uncertainty in medical image segmentation by adopting distinct probabilistic approaches. First, to improve aleatoric uncertainty modeling, Valiuddin et al. focus on incorporating normalizing flows into an ensemble setting, which allows for more accurate modeling of complex uncertainty distributions [16]. Chen et al. propose a scalable functional variational Bayesian neural network in which the prior and variational posterior are modeled as Gaussian processes, and variational inference is performed in function space. Their framework uses a content-aware U-Net and supports predictive inference in a single forward pass [17]. Additionally, Zhao et al. push the boundaries of Bayesian inference by incorporating Hamiltonian Monte Carlo (HMC), a powerful technique that enables sampling from complex posterior distributions in an efficient manner with a focus on improving the accuracy and efficiency of sampling [18]. Finally, Qin et al. introduce an approach utilizing a diffusion probabilistic model that provides a powerful way to model complex uncertainty distributions by learning to reverse a diffusion process [19].

#### 4.1.2. Monte Carlo Dropout (MC-Dropout)

Another prominent approach in uncertainty quantification leverages Monte Carlo dropout (MC-Dropout), a technique inspired by Bayesian inference that approximates posterior distributions by randomly dropping units during both training and inference. This method has gained considerable attention due to its simplicity and effectiveness in providing uncertainty estimates without using complex probabilistic models for generating uncertainty maps [20,21] or directly utilizing the uncertainty in a loss function to penalize uncertain regions [22].

To start, some of these methods were introduced to prevent the models from overfitting. Ghoshal et al. explored an ensemble of MC-Dropout models that mitigated issues related to model overfitting, leading to more robust predictions [23]. Other approaches combine MC-Dropout with other architectures to generate uncertainty maps. For instance, Krygier et al. extended the use of MC-Dropout in Bayesian CNNs; by generating probabilistic segmentation maps, quantifying voxel-wise uncertainty and applying thresholds to the probability maps, they derived multiple plausible segmentations. Next, they translated the segmentation samples into geometry for physics-based simulations and fitted statistical distributions to simulation results for efficient uncertainty approximation [7]. In addition, Zhang et al. also utilize MC-Dropout for uncertainty estimation. Their work is built upon utilizing both a regression branch that generates signed distance maps to capture geometric boundary information and a segmentation branch that generates prob maps for pixel-wise classification. Then, a cross-task consistency is performed to regularize consistency between the segmentation and regression outputs to enhance geometric understanding [24].

In a slightly different direction in semi-supervised learning, Zheng et al. incorporate MC sampling into a co-training framework. By leveraging the co-training method, they use two models trained on different views of the data, with MC-Dropout helping to quantify uncertainty in each model’s predictions [25]. Chen et al. employ a teacher–student model combined with MC-Dropout to capture uncertainty in a supervised learning setting. By transferring knowledge from a more confident teacher model to a student model that estimates uncertainty, they improve the overall performance of the model while maintaining a reliable measure of uncertainty [26].

Finally, Zhao et al. combine MC-Dropout with multi-dimensional models, where the framework combines 2D-CNNs to capture rich intra-slice details, 3D-CNNs to incorporate inter-slice spatial correlations, and 2.5D-CNNs to balance local and contextual information by considering adjacent slices. Each model shares soft-label predictions with the others, enabling mutual supervision. Then, with MC-Dropout, uncertainty maps are generated [27].

Some of the limitations of Bayesian uncertainty methods include being computationally intensive due to multiple forward passes or complex inference procedures [18,28]. Furthermore, scalability remains a key concern, particularly when dealing with high-dimensional medical data, where Bayesian frameworks can become impractical due to their computational and memory demands [29]. The MC-Dropout-based methods are an approximation and may yield suboptimal posterior estimates, limiting predictive accuracy [15,23]. Choosing appropriate priors is non-trivial, and poor prior assumptions can degrade model performance [6,10].

### 4.2. Deep Ensembles

Deep ensembles offer an effective method for capturing model uncertainty. This technique involves training multiple models independently and aggregating their predictions to estimate uncertainty. By utilizing different initializations or training paths for each model, deep ensembles can capture variations in model behavior, offering a more robust measure of uncertainty, especially in settings where training data may be limited. For an ensemble containing *S* independently trained models, each prediction can be written as $y^{\left(s\right)}=f_{\theta_{s}}\left(x\right)$. The ensemble predictive mean and variance are computed using Equations (1) and (2), respectively. Greater disagreement among the ensemble members results in greater estimated uncertainty.

To mitigate the challenges of training and storing multiple models, Buddenkotte et al. propose a scalable framework by calibrating linearly combined outputs from models trained with different loss weights, enabling efficient pseudo-label generation [29]. Wang et al. address uncertainty estimation by applying posterior bootstrap to nnU-Net for complex medical image segmentation tasks [8]. Alves et al. utilize uncertainty-guided self-learning with deep ensembles to iteratively expand the labeled set with high-confidence pseudo-labels [30], while Landgraf et al. explore ensemble distillation to compress uncertainty information from ensembles into a single, more efficient model [31].

Even with these advances, a fundamental limitation persists because deep ensembles require training and storing multiple models, a process that can be resource-intensive and impractical for large-scale applications [32].

### 4.3. Deterministic Methods

Deterministic methods are one of the important categories in which the computational burden of training the model is decreased. They estimate uncertainty without relying on stochastic sampling and use model outputs, confidence scores, evidential frameworks, or direct computations to quantify uncertainty. They often require only a single forward pass and can be easily integrated into existing pipelines. One approach of deterministic methods is to directly use the model’s output confidence scores, given by:

(6) $$u\left(x\right)=1-\max_{c}p\left(y=c|x\right).$$

where p(y=c|x) is the softmax probability of class *c*, and u(x) is the uncertainty score.

Another approach uses evidential deep learning, where the Dirichlet distribution models uncertainty:

(7) p(y|x)=Dir(α),

where α represents evidence, and the total uncertainty is:

(8) $$u\left(x\right)=\frac{C}{\sum_{i=1}^{C}\alpha_{i}}.$$

where *C* is the number of classes.

Some methods in this category utilize Dempster–Shafer theory, which offers a framework for combining uncertain and imprecise information, allowing for robust handling of uncertainty in decision-making scenarios [33,34,35]. By integrating belief functions with deep neural networks, these methods provide not only uncertainty estimates but also a means of modeling and fusing evidence from multiple sources to guide decisions [36]. Guo et al. introduce an uncertainty-guided CNN–transformer hybrid network, where the CNN encoder identifies uncertain regions in the input using normalized softmax entropy to calculate an uncertainty map, and the transformers focus on these high-uncertainty regions to establish global dependencies selectively, minimizing redundancy [37].

In a slightly different manner, Zou et al. integrate subjective logic with Dirichlet distributions by parameterizing voxel-wise predictions in a single forward pass, deriving the uncertainty mass directly from the Dirichlet parameters [38]. Meanwhile, Kushibar et al. propose a single network with multiple segmentation heads to enable both pixel-wise and image-level uncertainty estimation in a single forward pass [39]. Lourenço et al. quantify uncertainty by leveraging soft labels from multiple annotators, capturing variability in human-provided labels [40].

In another category, shape modeling is introduced to compute uncertainty deterministically. Tate et al. employ statistical shape modeling to account for shape variability, thus offering a robust method for quantifying segmentation uncertainty [41]. Similarly, Adiga et al. utilize anatomically aware autoencoders to capture anatomical variations, learning both deterministic and uncertain features within the data and thereby enabling anatomically guided uncertainty estimation [42].

In unsupervised learning, Li et al. propose an uncertainty self-learning approach that uses contrastive learning and Class Activation Maps (CAMs) to label high-saliency lesions and exclude uncertain regions from pseudo-labeling, allowing the network to learn their classification over time [43]. In semi-supervised settings, Luo et al. modify a 3D U-Net with a Pyramid Prediction Network and an Uncertainty Rectification Module to refine multi-scale segmentation, deriving uncertainty from scale discrepancies without extra forward passes [44]. Finally, Shi et al. introduce an uncertainty-weighted consistency framework that integrates Uncertainty-Weighted Prediction Consistency Training (UPCT) and Relation-Driven Consistency Training (RCT) with a U-Net–ResNet–50 encoder, enforcing consistency between decoders through KL-divergence and encoder-level perturbations [45].

Deterministic methods face several limitations, including their inability to fully capture uncertainty, particularly epistemic uncertainty, as they lack mechanisms to account for variations in model weights or explore multiple hypotheses [13]. They typically assume that training and testing data are drawn from the same distribution, making them susceptible to miscalibration and inaccurate predictions under domain shifts, such as differences in imaging scanners or anatomical variations, without adequately signaling uncertainty [28]. In medical imaging, where multiple valid segmentation labels often exist due to inter-radiologist variability, deterministic models tend to collapse all plausible segmentations into a single output, thereby losing information about the potential variability of segmentation boundaries [16]. Additionally, deterministic models generate binary or point-estimate probability maps in a single pass, requiring additional post-processing steps to derive voxel-wise confidence intervals or probability distributions [23]. Finally, their reliability is heavily dependent on model calibration, which can be challenging to achieve consistently.

### 4.4. Test-Time Data Augmentation

Test-time data augmentation (TTA) offers a practical approach to uncertainty quantification by applying various transformations to the input data during inference and observing the variability in the model’s predictions. This method allows us to estimate uncertainty without requiring any modifications to the model architecture, making it a convenient choice when working with pretrained models. For *S* test-time transformations, each prediction is mapped back to the original image space and expressed as $y^{\left(s\right)}=T_{s}^{-1}\left(f\left(T_{s}\left(x\right)\right)\right)$. The predictive mean and variance are then computed using Equations (1) and (2). In this setting, greater variance indicates greater sensitivity to the selected input transformations.

Lin et al. combine test-time augmentation with MC-Dropout, adding a fuzzy-uncertainty-based quality control algorithm [46], while Zhang et al. adopt rotation-variant TTA to handle spatial variability in segmentation [47]. Zimmer et al. leverage TTA for placenta segmentation by integrating classification and semantic segmentation [48], and Deng et al. employ multi-box prompts as TTA to refine object detection uncertainty [49]. Lastly, Yang et al. introduce spherical image projections as TTA, improving uncertainty quantification in settings with rotational and geometrical variability [50].

Some limitations exist in these methods, including increased computational cost during inference due to the multiple forward passes required for each transformation [51]. Additionally, TTA may not fully capture model uncertainty, as it primarily captures data uncertainty introduced by transformations rather than the variability arising from the model itself [52].

### 4.5. Hybrid Methods

Hybrid methods in uncertainty quantification integrate multiple components within one architecture or training pipeline, rather than relying on a single approach. This integration generally enhances robustness compared to using a single method. These methods face several limitations, including the inherent difficulty in hyperparameter tuning as each added component introduces new tuning dimensions [53]. Additionally, deploying them in production settings requires managing large, multi-part inference graphs and coordinating among diverse modules such as ensembles, transformers, and Bayesian sampling, which can hinder real-time performance or edge-based implementations [31]. Sahlsten et al. [54], along with Ghoshal et al. [23] and Mehta et al. [32], examine MC-Dropout within ensemble frameworks to enhance uncertainty estimation. Ziaee et al. propose UA-UNet, an uncertainty-guided teacher–student Residual U-Net that uses an ensemble of teacher models to generate reliable pseudo-labels for semi-supervised kidney tumor segmentation on KiTS23 [55]. Ren et al. integrate test-time augmentation with Monte Carlo dropout and deep ensembles within the nnU-Net framework [51], while Sikha et al. combine a Feature Pyramid Network with TTA, Monte Carlo dropout, and ensemble methods to improve uncertainty estimation robustness [52]. These examples highlight the complexity of balancing computational efficiency with methodological rigor in practice.

### 4.6. Foundation-Model-Based Uncertainty Quantification

Foundation-model-based uncertainty quantification is treated as a separate cross-cutting category because these approaches are defined primarily by their use of large pretrained and promptable segmentation models rather than by a single uncertainty estimation mechanism. A method is included in this category when it: (1) uses a segmentation foundation model, such as SAM or MedSAM, and (2) explicitly estimates, exploits, or mitigates uncertainty in the predicted segmentation, model adaptation, prompting process, or refinement procedure. Depending on the implementation, these methods may use deterministic confidence scores, stochastic sampling, prompt ensembles, test-time augmentation, or hybrid uncertainty estimation strategies.

The Segment Anything Model (SAM) [56] is a general-purpose vision foundation model based on a promptable segmentation framework. It accepts spatial prompts such as points, bounding boxes, and masks and was designed to support transfer to previously unseen segmentation tasks. Medical adaptations, including MedSAM [57,58], adapt this framework to clinical imaging domains.

Several recent approaches explicitly incorporate uncertainty into foundation-model-based medical segmentation. U-MedSAM introduces an uncertainty-aware loss with Sharpness-Aware Minimization to improve robustness across imaging modalities [59]. MedSAM-U uses a Multi-Prompt Adapter, Uncertainty-Guided Multi-Prompt module, and Uncertainty-Guided Prompt Adaptation to refine prompt-based predictions [60]. UP-SAM addresses epistemic and aleatoric uncertainty through supervised domain adaptation and unsupervised stochastic alignment [61]. FastSAM-3D Slicer incorporates uncertainty estimation into an interactive 3D segmentation environment to identify regions requiring refinement [62]. I-MedSAM combines implicit neural representations with uncertainty-guided sampling to iteratively refine segmentation boundaries [63], while UR-SAM uses predictive entropy and uncertainty-guided rectification to improve segmentation robustness [64].

These methods demonstrate that foundation models can support uncertainty-aware interactive segmentation, domain adaptation, prompt refinement, and quality control. However, their calibration and evaluation protocols remain insufficiently standardized, while their computational requirements, sensitivity to prompt selection, and generalization across clinical institutions require further investigation.

#### Prompt-Induced Uncertainty

Interactive foundation models introduce prompt-induced uncertainty, whereby changes in the location, number, or type of point, box, or mask prompts may produce different segmentations for the same image. Bounding-box tightness and the selection of positive and negative points may also affect the resulting segmentation. This source of uncertainty is particularly relevant in clinical settings because prompts may vary across users and levels of expertise.

Prompt-induced uncertainty can be evaluated by applying multiple plausible prompts and measuring disagreement among the resulting segmentations. Potential mitigation strategies include prompt ensembles, automatic prompt generation, prompt-quality assessment, uncertainty-guided prompt refinement, and requesting additional prompts when uncertainty remains high.

### 4.7. Other and Emerging Methods

This category includes uncertainty quantification approaches that do not fit the five primary mechanism-based families and are not based on segmentation foundation models. These methods commonly incorporate specialized anatomical, structural, topological, or representation-learning principles.

For example, Gupta et al. propose a topology-aware uncertainty framework based on probabilistic discrete Morse theory and graph-based structure modeling. Unlike pixel-wise confidence methods, their approach estimates uncertainty for topological structures such as vessel branches and connections [65]. Tate et al. incorporate shape priors and statistical modeling to represent anatomical variation [41]. Judge et al. use a contrastive loss to align image and segmentation latent representations, while a segmentation decoder reconstructs anatomically consistent segmentation maps from the learned latent space [66]. Other emerging methods use topology-aware representations, anatomical constraints, or specialized shape models to capture uncertainty that may not be represented adequately by conventional predictive distributions.

## 5. Metrics for Evaluating Uncertainty Estimation

An uncertainty estimation framework should not only generate meaningful uncertainty maps but also quantify how well these estimates correlate with actual segmentation errors or ambiguities. Incorporating multiple metrics provides a comprehensive view of how well a model’s uncertainty estimates align with its actual performance. Calibration metrics gauge whether overall confidence is appropriately scaled, while error–uncertainty correlations and threshold-based measures assess local reliability. Distributional metrics investigate whether predicted probabilistic distributions accurately capture segmentation variability, and qualitative assessments ensure clinical interpretability. Below, we summarize common metrics and evaluation procedures used to assess the quality of uncertainty estimation in medical image segmentation.

### 5.1. Diagnostic and Segmentation Performance Metrics

Metrics commonly used in diagnostic tasks primarily evaluate predictive accuracy or discrimination, whereas uncertainty evaluation metrics assess whether a model can reliably identify predictions that are likely to be incorrect. These two categories are related but not interchangeable. A model may achieve high segmentation accuracy while remaining poorly calibrated or overconfident in erroneous regions. Conversely, a model may successfully identify uncertain predictions without producing sufficiently accurate segmentations. A comparison of commonly used diagnostic and segmentation metrics, including their advantages, limitations, and applicability to uncertainty-aware medical image segmentation, is provided in Table 4.

Diagnostic metrics can be applied to uncertainty-aware segmentation by formulating uncertainty estimation as an error-detection task. In this formulation, an erroneous voxel, anatomical structure, or patient case is treated as a positive instance, while the corresponding uncertainty value is used as the error-detection score. Sensitivity then represents the proportion of segmentation errors correctly identified as uncertain, whereas specificity represents the proportion of correct predictions appropriately retained as confident. Precision indicates how frequently an uncertainty alert corresponds to an actual segmentation error, and the negative predictive value indicates how frequently a prediction classified as confident is actually correct.

Threshold-independent metrics can also be used in this setting. The area under the receiver operating characteristic curve (AUROC) measures the ability of uncertainty scores to rank erroneous predictions above correct predictions across different thresholds. However, voxel-wise segmentation errors are frequently rare relative to correctly classified background and foreground voxels. Consequently, AUROC may appear favorable under severe class imbalance. The area under the precision–recall curve (AUPRC) is generally more informative when the objective is to identify a relatively small number of erroneous or clinically problematic predictions.

Conventional segmentation metrics, including the Dice Similarity Coefficient, Intersection over Union (IoU), and boundary-distance measures such as the 95th-percentile Hausdorff distance (HD95), evaluate the quality of the predicted segmentation rather than the reliability of its uncertainty estimates. Dice and IoU quantify spatial overlap, while HD95 evaluates boundary disagreement and is particularly relevant when uncertainty is concentrated around anatomical boundaries. These metrics should therefore be reported alongside, rather than instead of, calibration and uncertainty-specific metrics.

Conventional diagnostic and segmentation metrics provide important information about predictive accuracy and error detection; however, they do not directly measure whether the estimated confidence or uncertainty is reliable. Therefore, these metrics should be complemented by uncertainty-specific measures. The following subsections discuss calibration, error–uncertainty correspondence, threshold-based evaluation, distributional goodness of fit, and qualitative assessment.

### 5.2. Calibration Metrics

Expected calibration error (ECE) measures how closely predicted confidence (or probability) corresponds to the true likelihood of correctness. ECE bins predictions according to their predicted probability and then computes the average difference between the predicted confidence and the empirical accuracy within each bin. Lower ECE values indicate better calibration [14,28].

Let the predictions be divided into *M* confidence bins, where $B_{m}$ denotes the set of predictions assigned to the *m*th bin. The Expected calibration error is defined as:

(9) $$\mathrm{ECE}=\sum_{m=1}^{M}\frac{|B_{m}|}{N}\left|\mathrm{acc}\left(B_{m}\right)-\mathrm{conf}\left(B_{m}\right)\right|,$$

where *N* is the total number of evaluated predictions, and the empirical accuracy and mean confidence of bin $B_{m}$ are respectively defined as:

(10) $$\mathrm{acc}\left(B_{m}\right)=\frac{1}{|B_{m}|}\sum_{i\in B_{m}}I\left({\hat{y}}_{i}=y_{i}\right),$$

(11) $$\mathrm{conf}\left(B_{m}\right)=\frac{1}{|B_{m}|}\sum_{i\in B_{m}}\max_{c}p_{i,c}.$$

Here, I(·) is the indicator function, ${\hat{y}}_{i}$ is the predicted class, $y_{i}$ is the ground-truth class, and $p_{i,c}$ is the predicted probability of class *c* for the *i*th pixel or voxel.

Reliability diagrams are a graphical tool for visualizing calibration. Predictions are grouped into intervals (e.g., 0–0.1, 0.1–0.2, …), and within each interval, the average predicted probability is plotted against the observed accuracy. Perfect calibration appears as a diagonal line, and deviations indicate miscalibration (over- or underconfidence) [29]. A reliability diagram is constructed by plotting $\mathrm{conf}(B_{m})$ against $\mathrm{acc}(B_{m})$ for each confidence bin. Unlike ECE, it is a graphical evaluation procedure rather than a single scalar metric.

The Brier score is the mean squared error between predicted probabilities and the ground-truth labels (zero or one). It captures both calibration (how well probabilities match outcomes) and refinement (the model’s ability to assign varying probabilities to different pixels/voxels). A lower Brier score indicates better overall calibration [10,13].

For a multiclass segmentation problem with *C* classes, the Brier score is defined as:

(12) $$\mathrm{BS}=\frac{1}{N}\sum_{i=1}^{N}\sum_{c=1}^{C}{\left(p_{i,c}-y_{i,c}\right)}^{2},$$

where $p_{i,c}$ is the predicted probability of class *c*, and $y_{i,c}$ is the corresponding one-hot-encoded ground-truth label. For binary segmentation, this expression reduces to:

(13) $$\mathrm{BS}=\frac{1}{N}\sum_{i=1}^{N}{\left(p_{i}-y_{i}\right)}^{2}.$$

### 5.3. Error–Uncertainty Correlation Metrics

An essential property of a good uncertainty estimate is that higher predicted uncertainty should correlate with higher segmentation errors, which can be obtained by Pearson or Spearman correlations. By computing the correlation coefficient between voxel-wise errors (e.g., absolute difference from ground truth) and the predicted uncertainty, one can measure the strength of this association. Higher positive correlation values indicate that the model is effectively capturing the locations of potential segmentation mistakes [7,23].

Let $u_{i}$ and $e_{i}$ denote the uncertainty and segmentation error of the *i*th prediction, respectively. Their Pearson correlation is:

(14) $$r_{u,e}=\frac{\sum_{i=1}^{N}\left(u_{i}-\bar{u}\right)\left(e_{i}-\bar{e}\right)}{\sqrt{\sum_{i=1}^{N}{\left(u_{i}-\bar{u}\right)}^{2}}\sqrt{\sum_{i=1}^{N}{\left(e_{i}-\bar{e}\right)}^{2}}}.$$

The Spearman correlation is equivalently computed using the ranks:

(15) $$\rho_{u,e}=r_{\mathrm{rank}(u),\mathrm{rank}(e)}.$$

Sparsification evaluates whether highly uncertain predictions also contain the greatest errors. Its performance can be measured using the Area Under the Sparsification Error curve:

(16) $$\mathrm{AUSE}=\int_{0}^{1}\left[E_{u}\left(r\right)-E_{\mathrm{oracle}}\left(r\right)\right]dr,$$

where $E_{u}\left(r\right)$ and $E_{\mathrm{oracle}}\left(r\right)$ are the retained errors after removing a fraction *r* of predictions according to uncertainty and true error, respectively. Lower AUSE indicates better uncertainty ranking [3,31].

### 5.4. Threshold-Based Metrics for Uncertainty Maps

In some workflows, setting a threshold on the uncertainty map yields a binary “uncertain region.” One can compare this uncertain region with areas of true segmentation errors by computing overlap metrics such as the Dice Similarity Coefficient. A higher Dice overlap suggests that uncertain areas align better with actual errors or ambiguous boundaries [22,40].

Let $U_{\tau }$ denote the region obtained by thresholding the uncertainty map at τ, and let *E* denote the true segmentation-error region. Their Dice overlap is:

(17) $${\mathrm{Dice}}_{U,E}=\frac{2|U_{\tau }\cap E|}{|U_{\tau }\left|+|E\right|}.$$

When the segmentation model outputs a probabilistic distribution (e.g., from Bayesian or ensemble methods), one can compute a voxel-wise or contour-wise prediction interval. The Coverage–Width metric evaluates whether the ground-truth segmentation is contained within a certain credible interval and how large that interval is on average. Ideal intervals have high coverage (capturing most of the ground truth) but remain narrow to avoid overestimating uncertainty [15].

For predictive lower and upper bounds $l_{i}$ and $r_{i}$, interval coverage and mean width are defined as:

(18) $$\mathrm{PICP}=\frac{1}{N}\sum_{i=1}^{N}I\left(l_{i}\leq y_{i}\leq r_{i}\right),$$

(19) $$\mathrm{MPIW}=\frac{1}{N}\sum_{i=1}^{N}\left(r_{i}-l_{i}\right).$$

High coverage and low width indicate reliable and informative intervals.

### 5.5. Distributional Goodness of Fit

For probabilistic or Bayesian methods, Negative Log-Likelihood (NLL) quantifies how well the predicted distributions over segmentations fit the ground-truth labels. Lower NLL indicates that the model is assigning high probability to the correct segmentation outputs, reflecting both accuracy and reliability of the uncertainty estimates [10,18].

For *N* predictions and *C* classes, the Negative Log-Likelihood is:

(20) $$\mathrm{NLL}=-\frac{1}{N}\sum_{i=1}^{N}\sum_{c=1}^{C}y_{i,c}\log p_{i,c},$$

where $p_{i,c}$ and $y_{i,c}$ are the predicted probability and ground-truth label for class *c*, respectively.

In methods that produce full parametric distributions (e.g., Dirichlet, Gaussian), the KL divergence can measure the difference between predicted distributions and empirical or ground-truth distributions (if available). While often used in training (e.g., variational inference), it can also serve as a metric to evaluate how faithful the model’s predicted distributions are to the true segmentation distribution [10,18].

The KL divergence between a reference distribution *P* and predicted distribution *Q* is:

(21) $$D_{\mathrm{KL}}\left(P∥Q\right)=\frac{1}{N}\sum_{i=1}^{N}\sum_{c=1}^{C}P_{i}\left(c\right)\log\left(\frac{P_{i}\left(c\right)}{Q_{i}\left(c\right)}\right).$$

Lower values indicate greater similarity between the two distributions.

### 5.6. Visual Inspection and Qualitative Analysis

While quantitative metrics are crucial, qualitative assessments—such as overlaying uncertainty maps on the original image or side-by-side comparison of predicted and ground-truth segmentations—provide insight into whether uncertainty hotspots correspond to anatomically ambiguous regions (e.g., tumor boundaries, low-contrast tissue interfaces). Clinicians often prefer visualizations that highlight “problematic” or “ambiguous” boundaries, making it easier to decide when additional review or imaging is necessary [34,35]. Visual inspection is a qualitative evaluation procedure and therefore does not have a single analytical expression. It should be used together with quantitative uncertainty evaluation metrics.

### 5.7. Comparative Summary of Uncertainty Evaluation Metrics

The uncertainty evaluation metrics discussed above assess different aspects of reliability, including calibration, error detection, spatial correspondence, distributional fit, and clinical interpretability. Table 5 summarizes their objectives, strengths, limitations, and suitability for medical image segmentation.

No individual metric provides a complete evaluation of uncertainty quality. Calibration metrics should therefore be combined with error-detection, spatial-localization, and distributional metrics. Qualitative visualization can further determine whether uncertainty estimates are anatomically meaningful and clinically interpretable. Overall, uncertainty evaluation should not rely on a single metric. Future studies should report at least one calibration metric, one uncertainty–error ranking or detection metric, and one spatial or qualitative assessment to provide a more complete and comparable evaluation.

## 6. Application Areas

Uncertainty quantification techniques are increasingly utilized in clinical contexts for medical imaging analyses. Medical segmentation is a vital step for localizing regions of interest, whether the pathological structures (tumors, lesions) or key anatomical features (organs, vessels). Yet, segmentation tasks have potential ambiguities arising from diverse imaging modalities, complex tissue appearances, and the inherent variability in patient anatomy.

Applications span a wide array of target anatomies from brain tumors [67,68] and cardiac structures [69,70] to lung nodules [71] and skin lesions [72]. For each, we highlight how uncertainty quantification methods are integrated into the segmentation pipeline, and why they are especially critical for that specific clinical or anatomical domain.

### 6.1. Brain Tumor Segmentation

Brain tumor segmentation, particularly with MRI data, benefits greatly from UQ due to the variability in tumor appearance and fuzzy boundaries between tumor subregions and healthy tissue. By quantifying uncertainty, segmentation models can better signal which tumor margins are ambiguous, potentially informing diagnosis and treatment strategies. In this context, Li et al. apply nnU-Net, showcasing how deterministic methods can still integrate uncertainty proxies, reinforcing segmentation reliability [34]. Yang et al. introduce the Spherical Projection-based U-Net (SPU-Net), using test-time augmentation to further assess model confidence during 3D tumor segmentation [50]. Sagar et al. use a variational autoencoder integrated with Bayesian neural networks to capture both epistemic and aleatoric uncertainties in tumor delineation [9].

### 6.2. Cardiac MRI Segmentation

Cardiac MRI segmentation involves delineating structures like the ventricles, myocardium, and atria. Given the heart’s dynamic nature and diverse patient presentations, UQ becomes crucial to identify ambiguous areas, especially at boundaries between different cardiac regions. In this context, Tsai et al. present Uncertainty-aware U-Mamba, a deterministic approach that nonetheless uses uncertainty cues to refine segmentation outcomes [73]. Guo et al. develop a CNN–transformer hybrid network that leverages uncertainty guidance [37]. Arega et al. apply a 3D U-Net with MC-Dropout, focusing on myocardial infarction segmentation and propagating uncertainty estimates [22].

### 6.3. Lung Nodule and Field Segmentation

Segmentation of lung structures and nodules in CT scans is inherently challenging due to the high variability in nodule shape, size, and appearance. UQ methods help in flagging regions where segmentation confidence is low, which is crucial for accurate disease detection and monitoring. In this context, Ding et al. propose FTransCNN to segment lung fields and related structures, incorporating fuzzy fusion techniques that implicitly consider uncertainty in segmentation boundaries [53]. Kohl et al. pioneer the Probabilistic U-Net method, setting a precedent for using Bayesian approaches to quantify segmentation uncertainty in lung imaging [15]. Carannante et al. use variational inference to handle uncertainties in lung CT, among other anatomies, improving boundary estimation in ambiguous lung regions [10].

### 6.4. Skin Lesion Segmentation

In dermatological imaging, precise segmentation of skin lesions is critical for accurate diagnosis and treatment planning. However, the variability of lesions demands careful interpretation of segmentation outputs, and UQ methods can highlight areas of low confidence where manual inspection or additional imaging might be warranted. In this context, Chen et al. evaluate a content-aware U-Net within a functional variational Bayesian framework on several medical image segmentation tasks, including skin-lesion segmentation using the ISIC 2018 dataset [17]. Li et al. explore deterministic methods for unsupervised skin lesion segmentation, discussing uncertainty proxies derived from model outputs [43]. Sikha et al. combine MC Dropout, ensemble methods, and test-time augmentation to improve segmentation accuracy and quantify uncertainties for skin lesions [52].

### 6.5. Prostate Segmentation

Prostate segmentation, often on MRI data, is crucial for planning interventions and monitoring disease progression. Due to the prostate’s complex boundaries and variability in tissue appearance, UQ helps ensure that segmentation outputs are trustworthy and highlights areas requiring expert review. In this context, Nguyen et al. utilize a 2D nnU-Net, discussing uncertainty interpretation even within a primarily deterministic framework [74]. Gaillochet et al. incorporate MC-Dropout in a U-Net architecture, using uncertainty to drive active learning in prostate segmentation [75]. Ghosal et al. apply MC-Dropout on a VGG–U-Net model, focusing on prostate tumor delineation and uncertainty quantification [76].

### 6.6. Polyp Segmentation (Colonoscopy)

Segmentation of polyps in colonoscopy images is critical for early detection and treatment of colorectal cancer. Due to variability in polyp shape, size, and texture, segmentation models can be uncertain in delineating exact boundaries, making uncertainty quantification particularly valuable. By identifying low-confidence areas, clinicians are alerted to review segments that may require further investigation or alternative imaging modalities. In this context, Valiuddin et al. employ a Probabilistic U-Net. Their approach provides segmentation masks for polyps along with uncertainty estimates, helping to identify ambiguous boundaries [16]. Zheng et al. propose CASF-Net, a CNN–transformer hybrid for polyp segmentation, using transformer-based attention mechanisms that also lend insight into uncertain predictions [77]. Yue et al. utilize a Pyramid Vision Transformer, providing detailed boundary segmentation with uncertainty cues that can highlight challenging regions due to variability in polyp appearance [78].

### 6.7. Others

Uncertainty quantification extends beyond primary areas like brain and cardiac segmentation to encompass abdominal organs, retinal imaging, and prognostic/multi-organ segmentation tasks, all of which share challenges such as anatomical variability, complex boundaries, and heterogeneous imaging conditions. In abdominal segmentation, methods by Wang et al. apply probabilistic ensembles to flag ambiguous liver and pancreas boundaries [79], while Ren et al. integrates UQ into 3D nnU-Net frameworks for complex head and neck cancers [51]. Gupta et al. apply a topology-aware uncertainty framework based on probabilistic discrete Morse theory and graph-based structure modeling to retinal vessels and other branching structures [65]. Prognostic and multi-organ segmentation approaches, such as those by Alves et al. leverage deep ensembles and hybrid frameworks to segment numerous organs simultaneously, using UQ to prioritize review of low-confidence regions, guide active learning, and improve model reliability across diverse anatomical regions [30]. Collectively, these studies demonstrate that a unified UQ strategy across varied and complex segmentation tasks not only enhances accuracy but also builds trust in automated clinical workflows.

### 6.8. Cross-Application Synthesis

Across clinical applications, the most useful form of uncertainty depends on the intended clinical decision. Boundary-level uncertainty is particularly relevant for tumors, lesions, and polyps, where inaccurate delineation may affect diagnosis or treatment planning. In contrast, structure- or case-level uncertainty may be more useful for quality control, automated referral, and multi-organ segmentation, where the primary objective is to identify predictions requiring expert review.

The preferred UQ strategy also depends strongly on workflow constraints. Offline, high-resolution 3D MRI and CT applications may justify Bayesian or ensemble approaches when uncertainty reliability is prioritized, whereas time-sensitive applications, such as colonoscopy and surgical guidance, favor calibrated single-pass methods. Consequently, UQ methods should be selected according to the required uncertainty granularity, available computational resources, acceptable inference time, and intended clinical action rather than segmentation accuracy alone.

## 7. Discussion

**Figure 4:**
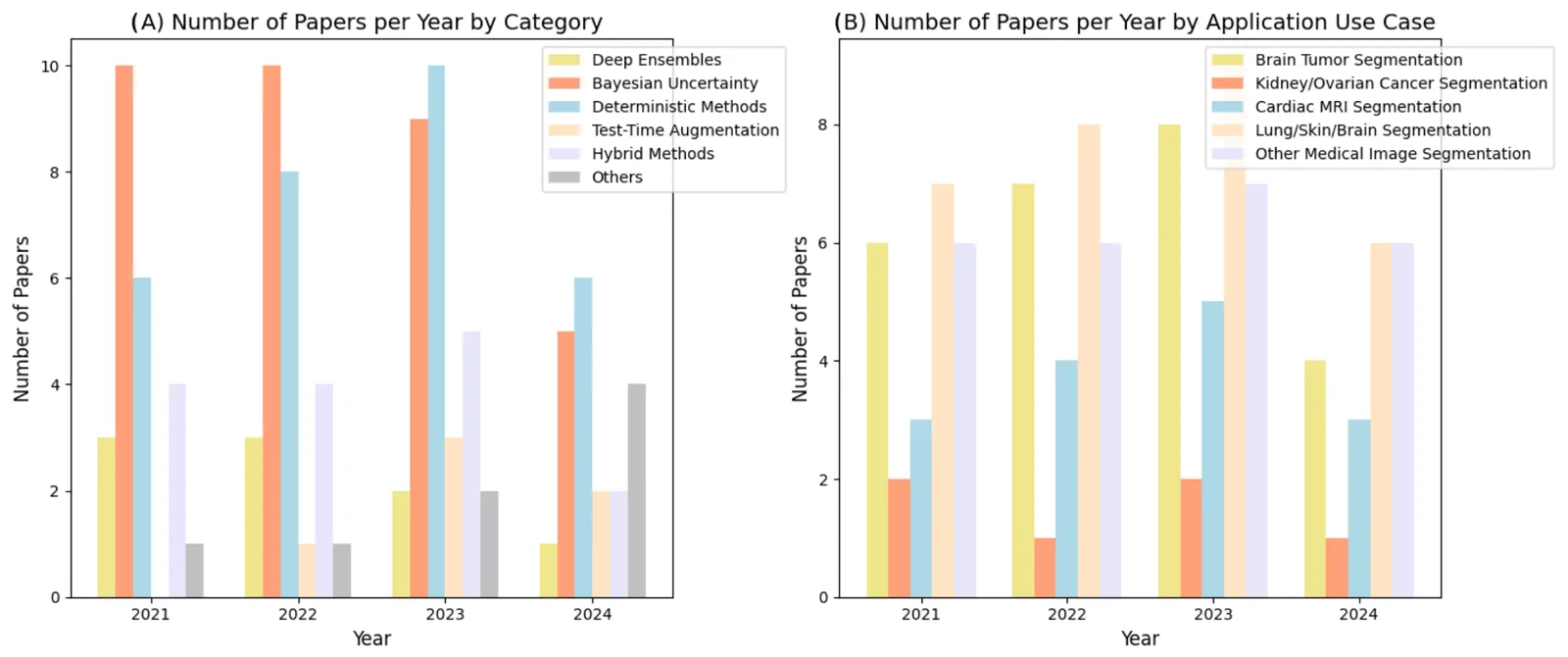
Number of papers annually in different medical image segmentation applications and by uncertainty estimation category.

Uncertainty quantification in medical image segmentation has seen significant advancements with the introduction of diverse methods to address both epistemic and aleatoric uncertainties. Bayesian approaches, such as Monte Carlo dropout (MC-Dropout) and Bayesian neural networks, have demonstrated their ability to estimate voxel-wise uncertainties by approximating posterior distributions through stochastic sampling. Deep ensembles, on the other hand, leverage multiple models with varying initializations or loss functions to capture global exploration capabilities and improve predictive reliability. Deterministic methods, such as those leveraging Dirichlet prior distributions or structured output space modeling, provide faster and computationally efficient alternatives by directly estimating uncertainty without the need for multiple forward passes or sampling. Test-time data augmentation complements these approaches by introducing input variability through transformations such as rotations or scaling and measuring predictive sensitivity across the augmented versions. Hybrid methods combining Bayesian, ensemble, and deterministic strategies have further enhanced robustness, especially in out-of-distribution scenarios. Figure 4A indicates the number of papers annually by each category of uncertainty estimation method.

Applications of uncertainty quantification span various tasks, including tumor segmentation, organ delineation, and boundary refinement. Methods such as pseudo-labeling with uncertainty guidance and active learning frameworks have proven effective in semi-supervised settings, where limited labeled data pose challenges. Additionally, innovations like soft labels, hierarchical latent spaces, and fusion models integrate uncertainty estimates into downstream tasks, such as treatment planning and diagnostic workflows. Although these methods address fundamental issues, such as annotation variability and prediction confidence, they also highlight the growing focus on scalable and interpretable uncertainty quantification strategies that integrate seamlessly into clinical applications. Figure 4B shows the number of papers annually by each application of medical image segmentation.

Table 6 summarizes the studies included in this narrative review with respect to their uncertainty estimation method, dataset, application, uncertainty category, and target imaging modality. The first column identifies the reviewed study, while the remaining columns report the model or estimation method, evaluated datasets, application area, UQ category, and corresponding imaging modalities.

Across the reviewed studies, most UQ approaches maintain competitive segmentation performance, while their main differences concern calibration, uncertainty quality, and computational requirements. Deep ensembles and hybrid methods commonly provide strong empirical uncertainty estimates but require substantial training and inference resources. MC-Dropout offers a practical compromise, whereas deterministic methods support efficient single-pass inference but may incompletely capture epistemic uncertainty. Test-time augmentation is model-agnostic but primarily measures sensitivity to input transformations. Reported numerical results are not directly comparable because the studies use different datasets, architectures, data splits, and evaluation metrics.

Despite significant advancements in uncertainty quantification for medical image segmentation, several challenges and limitations persist. One prominent issue is the computational cost associated with many state-of-the-art methods. Approaches like Monte Carlo dropout (MC-Dropout) and deep ensembles require multiple forward passes or training multiple models, making them computationally expensive, particularly for large-scale 3D datasets or high-resolution images (e.g., [13,23]). Deterministic methods offer computational efficiency, but they may lack the flexibility to model complex posterior distributions, potentially resulting in less accurate uncertainty estimates in challenging scenarios [11,13]. Furthermore, hybrid methods that combine multiple uncertainty quantification strategies often introduce additional complexity, which can hinder real-time applications in clinical settings.

Another critical challenge lies in the generalizability and interpretability of uncertainty estimates. Many methods demonstrate strong performance on specific datasets, such as BraTS or ACDC, but fail to generalize across diverse imaging modalities or tasks due to dataset biases or overfitting [28,40]. Moreover, while uncertainty maps can provide valuable insights, their clinical interpretability remains limited. Clinicians require uncertainty estimates to be actionable, yet most methods lack standardized visualization tools or thresholds for decision-making [33,34]. Addressing annotation variability is also a persistent limitation, as many datasets rely on single or inconsistent annotations, which can propagate errors during training and uncertainty estimation [36,40]. Lastly, the lack of standardized benchmarks for evaluating uncertainty quantification methods across datasets and tasks makes it difficult to compare methods and assess their clinical viability comprehensively.

From a practical perspective, clinical translation also requires careful consideration of computational cost, memory requirements, annotation burden, and workflow integration. Deep ensembles require multiple models to be trained and stored, while Monte Carlo dropout and test-time augmentation require repeated inference. These requirements may be impractical for high-resolution 3D images or time-sensitive clinical applications. Furthermore, methods that model annotation variability may require multiple expert annotations, increasing the time and cost of dataset development.

Uncertainty outputs must also be connected to clear clinical actions. Voxel-level uncertainty maps may support manual correction, whereas structure-level or case-level scores may be more appropriate for quality control, model abstention, or referral for expert review. However, poorly selected uncertainty thresholds may generate excessive alerts and reduce clinician trust. Clinical deployment therefore requires external validation, interpretable uncertainty representations, clinically meaningful thresholds, and integration with existing imaging and reporting workflows.

Several notable gaps in the literature hinder the full potential of uncertainty quantification in medical image segmentation. One major gap is the limited exploration of multimodal data integration for uncertainty estimation. Most studies focus on single-modality data, despite the potential of combining complementary information from modalities like CT, MRI, and PET to improve uncertainty robustness [33,54]. Additionally, while many methods address epistemic and aleatoric uncertainty, there is a lack of focus on modeling ambiguity caused by inter-annotator variability, which is common in medical imaging datasets [40,143]. Another underexplored area is the application of uncertainty in real-time decision support systems, where speed, interpretability, and clinical usability are critical. Although methods like test-time data augmentation and deterministic frameworks offer promising directions, their integration into time-sensitive workflows remains limited [11,13]. Furthermore, there is insufficient research on standardizing evaluation metrics for uncertainty quantification, leading to inconsistent reporting and difficulty in comparing methods across different datasets and tasks [28,34]. Segmentation foundation models, such as SAM and MedSAM, represent an emerging category of segmentation models. UQ for these models remains comparatively underexplored and insufficiently standardized, particularly regarding calibration, prompt variability, and external clinical validation. In addition, current UQ research remains focused on MRI and CT, with limited exploration of multimodal frameworks or understudied modalities like PET, ultrasound, and X-ray. While emerging adaptations of SAM to image segmentation and UQ (e.g., [59,60]) demonstrate cross-modal potential, UQ integration with multimodal images remains underexplored. This narrow scope impedes the development of robust, universally applicable clinical tools, particularly for modalities with unique noise profiles or dynamic acquisition challenges.

The future of uncertainty quantification in medical image segmentation lies in addressing the current challenges and leveraging emerging technologies to enhance robustness, scalability, and clinical applicability. One promising direction is the development of hybrid frameworks that integrate Bayesian, deterministic, and deep ensemble approaches to balance computational efficiency with accurate uncertainty estimation [13,34]. Additionally, the integration of multimodal data, combining information from CT, MRI, and other imaging modalities, can improve the reliability of uncertainty estimates, especially in complex tasks like tumor segmentation [33,54]. Semi-supervised and self-supervised learning methods that leverage unlabeled data through uncertainty-guided pseudo-labeling and active learning are also expected to grow, as these approaches address the scarcity of annotated medical datasets [26,143].

Future research should also focus on real-time and scalable solutions that can meet the demands of clinical environments. Techniques like single-pass uncertainty estimation and lightweight deterministic models could make uncertainty quantification feasible for time-sensitive applications such as surgical guidance or emergency diagnostics [11,40]. Standardizing evaluation metrics and creating publicly available benchmarks across various datasets and imaging modalities will also be critical for ensuring consistency and comparability in the field [28,34]. Moreover, enhancing clinical interpretability through intuitive visualizations and actionable uncertainty maps will enable better integration of uncertainty quantification into decision-making workflows. Lastly, emerging technologies, such as transformer-based architectures and diffusion models, offer new opportunities to improve the precision and robustness of uncertainty modeling, paving the way for their broader adoption in medical image analysis [18,65].

## 8. Conclusions

Uncertainty quantification has rapidly evolved into a cornerstone of modern medical image segmentation, addressing the critical need for reliable, interpretable models in high-stakes clinical settings. This survey has highlighted how different uncertainty estimation strategies ranging from Bayesian methods and deep ensembles to deterministic frameworks and test-time data augmentation offer complementary solutions for tackling both epistemic and aleatoric uncertainties. In doing so, these methods enhance segmentation robustness across diverse applications such as brain tumor delineation, cardiac structure identification, and polyp detection, among others. Their utility extends beyond segmentation boundaries, informing downstream tasks like treatment planning, disease monitoring, and clinical decision support.

Despite the considerable progress, several challenges and gaps remain. Computational overhead, domain shifts, annotation variability, and the lack of standardized benchmarks all limit the widespread adoption of these techniques in real-world clinical workflows. Future efforts must focus on developing more efficient, scalable approaches—potentially through hybrid frameworks that balance multiple uncertainty strategies—and on creating universally accepted evaluation protocols that allow cross-method comparisons. Likewise, enhancing clinical interpretability through intuitive uncertainty visualizations and actionable metrics will be pivotal for building trust among healthcare practitioners. As new technologies like large-scale foundation models (e.g., Segment Anything) and advanced probabilistic architectures continue to emerge, their integration with robust uncertainty quantification frameworks promises to reshape how medical imaging systems are designed, validated, and ultimately deployed for patient care.

## Figures and Tables

**Table 1:** Comparison of aleatoric and epistemic uncertainty.

Type	Aleatoric Uncertainty	Epistemic Uncertainty
**Nature**	Inherent randomness or noise in data	Lack of knowledge or insufficient training
**Reducibility**	Irreducible	Reducible with more data or better models
**Cause**	Data variability (noise, artifacts, anatomy)	Model limitations (domain shifts, sparse data)
**Example**	Blurred tumor edges in images	Model struggling with rare lesion types
**Management**	Robust models, noise-tolerant training	Data augmentation, active learning, Bayesian models

**Table 2:** Comparison of uncertainty quantification methods in medical image segmentation.

Method	Underlying Principle	Advantages	Limitations	Typical Application Scenarios
**Variational Inference and Probabilistic Models**	Approximate a posterior distribution over model parameters or latent variables using a tractable variational distribution. Depending on the formulation, these methods can model epistemic uncertainty, aleatoric uncertainty, or both.	Provide a principled probabilistic framework and can generate multiple plausible segmentations for ambiguous medical images.	Require specialized training objectives and assumptions about prior and variational distributions. The approximate posterior may not accurately represent the true posterior.	Suitable for tasks involving ambiguous boundaries, multiple valid segmentations, inter-annotator variability, and probabilistic shape modeling.
**Monte Carlo Dropout**	Keeps dropout active during inference and performs multiple stochastic forward passes. Variability among the resulting predictions is used primarily as an estimate of epistemic uncertainty.	Can be incorporated into existing CNN or U-Net architectures with relatively minor modifications and does not require training multiple independent models.	Requires multiple inference passes and provides only an approximation to Bayesian inference. Its performance depends on dropout placement, dropout rate, and the number of stochastic samples.	Suitable for voxel-wise uncertainty mapping, active learning, semi-supervised learning, and retrofitting uncertainty estimation into existing segmentation models.
**Deep Ensembles**	Train multiple models independently using different initializations, data subsets, or optimization paths. Uncertainty is estimated from disagreement among their predictions.	Generally provide robust uncertainty estimates, strong empirical calibration, and improved performance under distribution shifts.	Require training, storing, and evaluating multiple models, resulting in high computational, memory, and inference costs.	Suitable for safety-critical applications, out-of-distribution detection, and clinical systems in which uncertainty reliability is prioritized over computational efficiency.
**Deterministic Methods**	Estimate uncertainty in a single forward pass using confidence scores, evidential distributions, auxiliary prediction heads, learned uncertainty parameters, or anatomical and shape representations.	Provide fast inference, low computational cost, and relatively straightforward integration into real-time segmentation pipelines.	May not adequately capture epistemic uncertainty because model-parameter variability is not explicitly represented. Their reliability is strongly dependent on calibration and may decrease under domain shift.	Suitable for real-time clinical applications, resource-constrained systems, surgical guidance, and applications requiring rapid uncertainty estimation.
**Test-Time Data Augmentation**	Applies multiple clinically valid transformations to an input image and measures variability among predictions after transforming the outputs back to the original image space.	Is model-agnostic, requires no architectural modification, and can be applied to already trained or externally provided segmentation models.	Requires multiple inference passes and primarily captures sensitivity to input transformations rather than uncertainty in model parameters. Invalid transformations may produce misleading estimates.	Suitable when retraining or modifying the model is not possible and for tasks with meaningful geometric, rotational, or acquisition-related variability.
**Hybrid Methods**	Combine two or more uncertainty estimation strategies, such as Monte Carlo dropout, deep ensembles, test-time augmentation, Bayesian inference, or deterministic uncertainty estimation.	Can capture complementary sources of uncertainty and may provide greater robustness than an individual uncertainty estimation method.	Introduce substantial computational cost, implementation complexity, and additional hyperparameters. Their individual uncertainty components may also be difficult to interpret separately.	Suitable for research settings and safety-critical applications requiring comprehensive uncertainty estimation, particularly under domain shift or out-of-distribution conditions.
**Foundation-Model-Based Methods**	Use large pretrained and promptable segmentation models, such as SAM or MedSAM, together with uncertainty estimation, prompt refinement, stochastic sampling, or uncertainty-guided adaptation.	Support interactive segmentation, broad transferability, prompt-based refinement, and adaptation across multiple imaging tasks.	Can be sensitive to prompt selection, computationally demanding, difficult to calibrate, and insufficiently validated across clinical institutions.	Suitable for interactive medical segmentation, uncertainty-guided prompting, quality control, domain adaptation, and human-in-the-loop workflows.
**Other and Emerging Methods**	Use specialized mechanisms such as statistical shape models, topology-aware representations, contrastive learning, anatomical constraints, or other representation-learning approaches.	Can incorporate anatomical, structural, or task-specific knowledge that is not explicitly represented by conventional uncertainty estimation methods.	Form a heterogeneous category with limited standardization, making direct comparison, validation, and generalization difficult.	Suitable for applications involving anatomical consistency, topology preservation, structural constraints, and specialized clinical requirements.

**Table 3:** Unified cross-method comparison of uncertainty quantification approaches.

Method Category	UQ Quality	Computational Cost and Scalability	Calibration	Interpretability	Clinical Applicability
**Bayesian Methods**	High potential for capturing epistemic uncertainty and aleatoric uncertainty when explicitly modeled	Moderate to high cost due to stochastic sampling or complex inference; scalability may be limited for high-resolution 3D images	Moderate to high, but dependent on prior selection and posterior approximation	Moderate; probabilistic outputs are meaningful, although posterior behavior may be difficult to explain	Suitable when probabilistic reliability is prioritized over inference speed
**Deep Ensembles**	Generally high because disagreement among independently trained models captures model variability	High training, storage, memory, and inference cost; scalability decreases as ensemble size and image resolution increase	Often strong empirically, including under moderate distribution shift	Moderate; prediction disagreement is intuitive, but individual model contributions may be difficult to interpret	Suitable for safety-critical or offline workflows with sufficient computational resources
**Deterministic Methods**	Moderate and method-dependent; epistemic uncertainty may be incompletely represented	Low cost because uncertainty is commonly estimated using one model and one forward pass; generally highly scalable	Variable and strongly dependent on the training objective and calibration procedure	Moderate to high when uncertainty is represented using confidence or evidential scores	Suitable for real-time and resource-constrained workflows when calibration has been validated
**Test-Time Data Augmentation**	Moderate; primarily captures sensitivity to input transformations rather than complete model uncertainty	Moderate to high inference cost because several transformed inputs are evaluated; scalable without retraining, but slower with more augmentations	Variable and dependent on the selected transformations and aggregation method	Moderate because prediction variation across transformations is relatively intuitive	Suitable when clinically valid transformations are available and the trained model cannot be modified
**Hybrid Methods**	Potentially high because complementary sources of uncertainty can be combined	High to very high cost due to multiple models, sampling procedures, or augmentation strategies; generally difficult to scale	Potentially strong, but dependent on the calibration of all combined components	Low to moderate because the contributions of individual components may be difficult to separate	Suitable for research and safety-critical applications, but less practical for real-time deployment
**Foundation-Model-Based Methods**	Method-dependent; can capture model, data, and prompt-related uncertainty depending on the UQ mechanism	Moderate to high because of large model size, repeated prompting, or multiple inference passes; deployment may require substantial memory	Currently insufficiently standardized and sensitive to model adaptation and prompt selection	Moderate; prompt disagreement can be intuitive, but internal model behavior is difficult to explain	Promising for interactive segmentation, although standardized prompting, calibration, and workflow validation are still required

**Table 4:** Comparison of common diagnostic and segmentation metrics and their applicability to uncertainty-aware medical image segmentation.

Metric	Primary Objective	Applicability to Uncertainty Evaluation	Main Limitation
**Accuracy**	Measures the overall proportion of correct predictions	Provides a general measure of voxel-, structure-, or case-level predictive performance	Can be misleading when the foreground class or segmentation errors are rare
**Sensitivity/** **Recall**	Measures the proportion of positive instances correctly detected	Measures the proportion of segmentation errors correctly identified as uncertain when errors are treated as the positive class	Does not account for correct predictions that are incorrectly flagged as uncertain
**Specificity**	Measures the proportion of negative instances correctly identified	Measures the proportion of correct predictions appropriately retained as confident	Can be dominated by the large number of correctly classified background voxels
**Precision**	Measures the reliability of positive predictions	Measures the proportion of uncertainty alerts that correspond to actual segmentation errors	Depends strongly on the prevalence of segmentation errors
**Negative** **Predictive** **Value**	Measures the reliability of negative predictions	Measures the proportion of predictions classified as confident that are actually correct	Depends on error prevalence and the selected uncertainty threshold
**Dice Similarity** **Coefficient**	Measures spatial overlap between predicted and reference segmentations	Evaluates segmentation accuracy and can also measure the overlap between thresholded uncertainty regions and actual segmentation errors	Requires thresholding and does not measure probabilistic calibration
**Intersection** **over Union**	Measures spatial overlap relative to the union of predicted and reference regions	Evaluates segmentation accuracy or the correspondence between uncertain and erroneous regions	Does not evaluate confidence calibration or uncertainty ranking
**AUROC**	Measures discrimination across all possible thresholds	Evaluates whether uncertainty scores rank erroneous predictions above correct predictions	May appear overly favorable when segmentation errors are highly imbalanced
**AUPRC**	Measures the trade-off between precision and recall across thresholds	Is particularly suitable for detecting relatively rare erroneous voxels, anatomical structures, or patient cases	Its value depends on the prevalence of segmentation errors
**HD95**	Measures disagreement between predicted and reference boundaries	Evaluates clinically important boundary errors and whether high uncertainty occurs near inaccurate anatomical boundaries	Does not measure calibration and may be influenced by isolated boundary errors

**Table 5:** Comparison of metrics for evaluating uncertainty estimation in medical image segmentation.

Metric	Objective	Strengths	Limitations	Suitability
**Expected Calibration Error**	Measures the difference between predicted confidence and observed accuracy	Simple, intuitive, and widely used for evaluating calibration	Sensitive to the number and definition of confidence bins	Suitable for evaluating global, class-wise, or voxel-wise calibration
**Reliability Diagram**	Visualizes the relationship between predicted confidence and empirical accuracy	Clearly identifies overconfidence and underconfidence	Depends on binning and does not provide a single numerical value	Useful for visually assessing model calibration alongside ECE
**Brier Score**	Measures the squared difference between predicted probabilities and ground-truth labels	Evaluates both probabilistic accuracy and calibration using a proper scoring rule	Does not clearly separate calibration error from segmentation accuracy	Suitable for binary and multiclass voxel-wise probabilistic predictions
**Pearson Correlation**	Measures the linear relationship between predicted uncertainty and segmentation error	Simple to compute and easy to interpret	Does not capture nonlinear relationships and may be sensitive to outliers	Suitable for evaluating linear uncertainty–error correspondence
**Spearman Correlation**	Measures the monotonic relationship between uncertainty and segmentation error	Less dependent on the numerical scale of uncertainty values	Evaluates ranking rather than probabilistic calibration	Suitable for comparing uncertainty rankings across different methods
**AUSE**	Measures how closely uncertainty-based error ranking approaches an oracle ranking	Evaluates whether highly uncertain predictions contain the greatest errors	Depends on the selected error measure and sparsification procedure	Suitable for selective prediction, quality control, and automated referral systems
**Uncertainty–Error Dice**	Measures spatial overlap between thresholded uncertainty and actual error regions	Provides an intuitive measure of uncertainty localization	Requires selecting an uncertainty threshold	Suitable for voxel-wise error localization and boundary uncertainty evaluation
**Coverage–Width**	Evaluates whether predictive intervals contain reference values while remaining narrow	Balances interval reliability and informativeness	Less straightforward for categorical segmentation masks	Suitable for probabilistic contours, boundaries, and shape representations
**Negative Log-Likelihood**	Measures the probability assigned to the ground-truth segmentation	Strongly penalizes confident incorrect predictions and is a proper scoring rule	Sensitive to extremely small predicted probabilities	Suitable for probabilistic, Bayesian, and ensemble-based segmentation models
**KL Divergence**	Measures the difference between predicted and reference probability distributions	Directly compares complete probability distributions	Requires a reliable reference distribution, which may not always be available	Suitable when multiple annotations or empirical segmentation distributions exist
**Qualitative Visualization**	Assesses whether uncertainty is concentrated in clinically ambiguous or erroneous regions	Provides intuitive and anatomically meaningful interpretation	Subjective and difficult to standardize across observers	Suitable as a complementary assessment for clinical interpretation

**Table 6:** Categorization of the reviewed studies by uncertainty estimation method, dataset, application, uncertainty-capturing approach, and target imaging modality.

Paper	Estimation	Dataset	Use Case	Category	Imaging Modality
Fuchs et al. [28]	Multi-headed variational U-Net	BraTS 2018 [80]	Brain tumor segmentation (supervised)	Hybrid (variational inference, deep ensembles)	3D MRI
Buddenkotte et al. [29]	Ensemble of U-Net with Resnet decoder	Kits21 [81] and Ovarian Cancer (private dataset)	Kidney and ovarian cancer segmentation (supervised)	Deep ensembles	3D CT & MRI
Sagar et al. [9]	Variational autoencoder with BNNs	BraTS 2018 [80]	Brain tumor segmentation (supervised)	Bayesian uncertainty (variational inference)	3D MRI
Carannante et al. [10]	Variational inference	Lungs CT [82], Hippocampus MRI [83], BRATS [84]	Segmentation of brain, hippocampus, and lungs (supervised)	Bayesian uncertainty (variational inference)	3D CT & MRI
Ghoshal et al. [23]	MC dropout and deep ensembles	Kaggle Data Science Bowl Challenge [85]	Medical image segmentation, and disease detection (supervised)	Hybrid—MC-Dropout and deep ensembles	2D microscopy images
Lourenço-Silva et al. [40]	U-Net architecture with an EfficientNet-B0	MICCAI 2021 QUBIQ Challenge [86]	Medical image segmentation (supervised)	Deterministic methods	3D CT
Yang et al. [87]	Multi-decoder U-Net	MICCAI 2020 QUBIQ Challenge [88]	Medical image segmentation (supervised)	Deterministic methods	3D CT
Krygier et al. [7]	Bayesian CNNs and MC dropout	Human Torso [89]	Segmentation of spine/aorta (supervised)	Bayesian uncertainty (MC-Dropout, Bayesian CNN)	2D CT
Wang et al. [79]	Probabilistic U-Net, Matting Network	LIDC-IDRI [90], QUBIQ [88], ISIC 2018 [91]	Lung, skin, brain segmentation (semi-supervised)	Bayesian uncertainty	3D CT & MRI & 2D dermoscopy
Jones et al. [11]	3D U-Net	Connectome [92]	Brain image segmentation (supervised)	Bayesian uncertainty	3D MRI
Ghesu et al. [33]	Deep convolutional neural network (CNN)	ChestX-Ray8 [93], PLCO [94]	Medical image assessment and segmentation (brain, abdominal, chest)	Deterministic methods	3D CT and X-ray
Kushibar et al. [39]	U-Net with multiple segmentation heads	Breast Mass Segmentation [95], Cardiac MRI Segmentation [70]	Breast, cardiac image segmentation (supervised)	Deep ensembles	3D MRI and 2D breast imaging
Li et al. [34]	nnU-Net	BraTS 2020 [67]	Brain tumor segmentation (supervised)	Deterministic methods	3D MRI
Tate et al. [41]	Statistical modeling of 9 manual segmentations	EDGAR [96]	Medical image segmentations (ECGI study) (supervised)	Others (Polynomial Chaos Expansion (PCE))	3D CT
Mehta et al. [32]	MC-Dropout, deep ensembles, dropout ensemble	MS T2 Lesion Segmentation [97], BraTS 2020 [67], ADNI [98]	Cardiac ventricular and pericardial surface segmentation (supervised)	Hybrid methods (MC-Dropout, Deep Ensembles)	3D MRI
Yang et al. [50]	Spherical projection-based U-Net (SPU-Net)	BraTS 2020 [67]	Brain tumor segmentation (glioma) (supervised)	Test-time data augmentation	3D MRI
Rajaraman et al. [20]	VGG16-based U-Net (MC dropout)	Shenzhen TB (2014) [99]	Medical image segmentation (chest) (supervised)	Bayesian uncertainty (MC-Dropout)	X-ray
Lambert et al. [100]	Anisotropic hybrid U-Net (AHUNet)	ATLAS 2023 [101]	Liver tumor segmentation (supervised)	Deterministic methods	3D MRI
Ghosal et al. [76]	MC-Dropout (MCD)-enabled Bayesian VGG–U-Net	Gleason 2019 Grand Challenge [102]	Prostate tumor segmentation (supervised)	Bayesian uncertainty (MC-Dropout)	2D H&E dye stained prostate tissue
Zhao et al. [13]	Ensemble of checkpoints (nnU-Net)	ACDC (in-domain) [69], M&M (out-of-domain) [70]	Cardiac MRI segmentation (supervised)	Bayesian uncertainty (Variational Inference)	3D MRI
Alshehhi et al. [103]	GAN-based U-Net	BraTS 2017 [104], 2018 [80], 2019 [105]	Brain tumor segmentation (supervised)	Bayesian uncertainty	3D MRI
Faghani et al. [106]	Various 3D CNNs + MCD	BraTS 2020 [67], CheXpert [107], LIDC-IDRI [71]	Brain, lung, cervical cancer, pancreas segmentation (supervised)	Hybrid methods (MC-Dropout, deep ensembles)	3D MRI, 2D chest X-ray, and 3D CT
Zhang et al. [47]	ConvNets with MCD	LUNA16 [108], LiTS17 [109]	Lung and liver segmentation (supervised)	Bayesian uncertainty (MC-Dropout)	3D CT
Arega et al. [22]	3D U-Net + MCD	EMIDEC [110]	Cardiac MRI segmentation (supervised)	Bayesian uncertainty (MC-Dropout)	3D MRI
Lin et al. [46]	Segmentation-quality control (no new CNN)	ISIC 2018 [91], DSB 2018 [85], JSRT [111], INBreast [112], MoNuSeg [113]	Skin, nuclei, lung, breast, and cell segmentation (supervised)	Hybrid methods (MC-Dropout, test-time data augmentation)	2D dermoscopy, microscopy/ histopathology, chest X-ray, and mammography
Zheng et al. [25]	U-Net + MCD	ACDC [69], SCGM [114], Spleen [115]	Cardiac MRI, Spinal Cord, spleen segmentation (semi-supervised)	Bayesian uncertainty (MC-Dropout)	3D MRI and 3D CT
Chen et al. [26]	U-Net + MCD	ACDC [69], SCGM [114], Spleen [115]	Cardiac MRI, spinal cord, spleen segmentation (semi-supervised)	Bayesian uncertainty	3D MRI and 3D CT
Zhang et al. [116]	Focus on UQ in TMS (no new model)	Brain MRI (private dataset)	Brain image segmentation (computational simulation and uncertainty analysis study)	Bayesian uncertainty (MC-Dropout)	3D MRI
Mojiri Forooshani et al. [21]	Bayesian 3D U-Net (MCD)	White Matter Hyper intensity [117], Multi-Center MRI (private)	White matter segmentation (supervised)	Bayesian uncertainty (MC-Dropout)	3D MRI
Chlebus et al. [118]	3D Anisotropic U-Net	LiTS [109]	Liver image segmentation (supervised)	Bayesian uncertainty (MC-Dropout)	3D CT
Valiuddin et al. [16]	Probabilistic U-Net	LIDC-IDRI [71], Kvasir-SEG [119]	Lung nodule and polyp segmentation (supervised)	Bayesian uncertainty	3D CT and 2D endoscopy
Chen et al. [17]	Content-aware U-Net with a functional variational Bayesian neural network and Gaussian-process prior and posterior distributions	ISIC 2018 [91], Lung CT Segmentation, CVC-ClinicDB [120], DRIVE [121]	Skin-lesion, lung, polyp, and retinal-vessel segmentation (supervised)	Bayesian uncertainty (functional variational inference)	2D dermoscopy, 3D CT, 2D endoscopy, and 2D retinal fundus
Kohl et al. [15]	Probabilistic U-Net (U-Net + CVAE)	LIDC-IDRI [71], Cityscapes [122]	Semantic image segmentation (supervised)	Bayesian uncertainty	3D CT and 2D RGB street-scene images
Baumgartner et al. [3]	PhiSeg	LIDC-IDRI [71]	Lung, prostate image segmentation (supervised)	Bayesian uncertainty	3D CT
Zhao et al. [18]	HMC with U-Net	ACDC [69], UK Biobank [123]	Cardiac MRI, quantitative MRI segmentation, and domain adaptation (supervised)	Bayesian uncertainty (Hamiltonian Monte Carlo)	3D MRI
Sahlsten et al. [54]	3D residual U-Net w/MCD, ensembles	HECKTOR 2021 [124]	Oropharyngeal cancer gross tumor volume segmentation (supervised)	Hybrid methods (MC-Dropout, deep ensemble)	3D PET/CT
Gupta et al. [65]	U-Net with GNN and probabilistic discrete Morse theory	DRIVE [121], ROSE [125], ROADS [126], PARSE [127]	Retinal-vessel, retinal OCTA-vessel, road-network, and pulmonary-artery segmentation (supervised)	Other and emerging methods (topology-aware uncertainty)	2D retinal fundus, 2D OCTA, 2D aerial images, and 3D CT
Wang et al. [8]	Ensemble of nnU-Net models	LiTS [109]	Liver tumor segmentation (supervised)	Bayesian uncertainty	3D CT
Alves et al. [30]	Deep ensemble of 3D nnU-Net	Total Segmentator [128]	Medical image segmentation (semi-supervised)	Deep ensembles	3D CT
Liu et al. [129]	U-Net w/3 decoders	RETOUCH [130]	Multiclass retinal fluid segmentation (semi-supervised)	Hybrid methods (MC-Dropout, self-ensembling)	2D optical coherence tomography (OCT)
Huang et al. [36]	Light U-Net (LUNet)	BraTS 2018 [80], BraTS 2019 [105]	Brain tumor segmentation (semi-supervised)	Deterministic methods	3D MRI
Zou et al. [38]	V-Net or attention U-Net	BraTS 2019 [105]	Brain tumor segmentation (supervised)	Deterministic methods	3D MRI
Judge et al. [66]	Encoder–decoder (contrastive + anatomical priors)	CAMUS [131], Shenzhen [99], HMC-QU (private)	Cardiac ultrasound, myocardium, lung segmentation (supervised)	Other methods (contrastive learning based)	2D echocardiography and chest X-rays
Adiga et al. [42]	3D U-Net + DAE	LGE-MRI [132], Abdominal CT [133]	Left atrium, abdominal multi-organ (semi-supervised)	Deterministic methods	3D MRI & CT
Yue et al. [78]	Pyramid Vision Transformer	Kvasir-SEG [119], CVC-ClinicDB [120], CVC-ColonDB [134], CVC-T [135], ETIS-LaribPolypDB (private)	Polyp image segmentation (supervised)	Deterministic methods	Endoscopic
Huang et al. [136]	Uncertainty-based Active Learning (AL) + Bayesian U-Net	CBCT [136]	Medical image segmentation (supervised)	Bayesian uncertainty (MC-Dropout)	3D CT
Hu et al. [35]	U-Net + Dirichlet distribution	Liver tumor (private)	Liver tumor segmentation (supervised)	Deterministic methods	3D CT
Jin et al. [137]	Pseudo-mask guided feature aggregation	MoNuSeg [113], CRAG [138]	Nuclei and gland segmentation (semi-supervised)	Deterministic methods	2D histopathology
Guo et al. [37]	Uncertainty-guided CNN–transformer hybrid network	Synapse [101], ACDC [69], ISIC 2018 [91]	Abdominal organ, cardiac MRI, skin lesion segmentation (supervised)	Others (deterministic with CNNs and transformers)	3D MRI & CT, 2D dermoscopic
Li et al. [43]	ResNet50 + fully convolutional network	ISIC 2017 [72], ISIC 2018 [91], PH2 [139]	Skin lesion segmentation (unsupervised)	Deterministic methods	2D dermoscopic
Wu et al. [140]	2D U-Net(M&MS, FB) or 3D U-Net(FeTA)	M&M [70], FeTA [141], FB (private)	Cardiac structure, fetal brain, fetal tissue segmentation (unsupervised)	Hybrid methods (MC-Dropout, deep ensembles)	3D MRI
Luo et al. [44]	3D Unet (pyramid predictions, uncertainty rectif.)	Panacreas-NIH [142], BraTS 2019 [105], NPC-MRI (private)	Nasopharyngeal Carcinoma (NPC), whole brain tumor, pancreas segmentation (semi-supervised)	Deterministic methods	3D MRI & CT
Wu et al. [143]	Diagnosis-First Segmentation (DiFF)	REFUGE-2 [144], TNMIX [145,146], ISIC [147]	Optic Disc/Optic Cup (OD/OC), thyroid nodule, skin lesion segmentation (supervised)	Deterministic methods	2D ultrasound & dermoscopic & fundus
Xu et al. [148]	3D U-Net	BraTS 2020 [67], LA 2018 [149]	Brain tumor, left atrial segmentation (semi-supervised)	Bayesian method (MC-Dropout)	3D MRI
Lu et al. [150]	V-Net (Mean-Teacher)	LA 2018 [149]	Left atrium segmentation (semi-supervised)	Deterministic methods	3D MRI
Zhao et al. [27]	2D, 2.5D, 3D UMM-Net (MCD)	IBSR [151], BraTS 2020 [67], MeniSeg [152]	Brain tumor segmentation (supervised)	Bayesian method (MC-Dropout)	3D MRI
Zhang et al. [24]	V-Net (MCD-based)	LA [149], BraTS 2019 [105]	Left atrium and brain tumor segmentation (semi-supervised)	Bayesian method (MC-Dropout)	3D MRI
Lu et al. [153]	V-Net	Pancreas-CT [154], LA [149], BraTS 2019 [105]	Pancreas, left atrium, brain tumor segmentation (semi-supervised)	Deterministic Methods	3D MRI & CT
Shi et al. [45]	U-Net w/ResNet-50 encoder, multi-decoders	NNPC (private), ACDC [69]	Nasopharyngeal Carcinoma (NPC), cardiac (semi-supervised)	Deterministic methods	3D CT
Li et al. [155]	U-Net/OM-VAE	Source_Prim, Source_Sec	Prostate gland segmentation (supervised)	Bayesian uncertainty	2D CT
Zimmer et al. [48]	TUNet, MTUNet, TMTUNet (+MCD)	Custom Placenta [48]	Placenta segmentation (supervised and multi-task learning)	Bayesian method (MC-Dropout)	3D Ultrasound
Gaillochet et al. [75]	4-layer U-Net + MCD	PROMISE12 [156], Medical Segmentation Decathlon Hippocampus [157]	Prostate, hippocampus segmentation (supervised)	Bayesian method (MC-Dropout)	3D MRI
Ding et al. [53]	FTransCNN (ResNet50 + transformer + fuzzy fusion)	Lung Segmentation (private), Kvasir-Seg [119]	Lung, gastrointestinal polyp, kidney, breast ultrasound segmentation (supervised)	Deterministic methods	2D CT
Deng et al. [49]	SAM-U (Segment Anything Model + MC sims)	REFUGE [158]	Optic disc and optic cup segmentation (unsupervised)	Test-time augmentation	2D Fundus
Shen et al. [159]	DeepLabv3+ (2D)/VNet (3D)	ISIC [91], Kvasir-SEG [119], CVC-ClinicDB [160], Left Atrial [149]	Skin, polyp, left atrium segmentation (semi-supervised)	Deterministic methods	2D dermoscopic & endoscopic, 3D MRI
Zheng et al. [77]	CASF-Net (CNN + transformer)	Kvasir-SEG [119], CVC-ClinicDB [160], EndoScene [161], ISIC-2018 [91], GLAS [162]	Polyp, skin lesion, gland segmentation (supervised)	Deterministic methods	2D gastrointestinal, 2D dermoscopic, 2D histology
Xu et al. [163]	Ambiguity-Consensus Mean-Teacher (AC-MT)	Left Atrium (LA) [149], BraTS 2019 [105]	Left atrium, brain tumor segmentation (semi-supervised)	Bayesian method (MC-Dropout)	3D MRI
Tsai et al. [73]	Uncertainty-aware U-Mamba (U-Net-based)	ACDC [69]	Cardiac MRI segmentation (supervised)	Deterministic methods	3D MRI
Ren et al. [51]	3D nnU-Net (+TTA, MCD, Phiseg, Ensembles, etc.)	Head and Neck Cancer (HNC; private)	Primary tumor, lymph node metastases segmentation (supervised)	Hybrid methods (MC-Dropout, deep ensembles, test-time augmentation)	3D MRI
Sikha et al. [52]	FPN (ResNet50) MCD, ensemble, TTA	HAM10000 [164]	Skin lesion segmentation (supervised)	Hybrid methods (MC-Dropout, deep ensembles, test-time augmentation)	2D dermoscopy
Huang et al. [165]	2D U-Net, 3D VNet	ISIC [91], Kvasir-SEG [119], CVC-ClinicDB [160], Left Atrial [149]	Skin lesion, colon polyp, left atrium segmentation (semi-supervised)	Deterministic methods	2D dermoscopic & endoscopic, 3D MRI
Zhao et al. [6]	Probabilistic-SwinUNETR	Real-World Epicardial Fat (private)	Epicardial Adipose Tissue (EAT) segmentation (supervised)	Bayesian uncertainty	3D MRI
Nguyen et al. [74]	2D nnU-Net	ProstateX [166]	Prostate Whole Gland (WG) segmentation (supervised)	Deterministic methods	3D MRI
Qin et al. [19]	Diffusion Probability Model (DPM) (U-Net-based)	BraTS 2020 [67], BraTS 2021 [68]	Brain tumor segmentation (supervised)	Other and emerging methods—diffusion probabilistic model	3D MRI
Wang et al. [59]	SAM with uncertainty-aware loss and SharpMin	CVPR24 MedSAM on Laptop competition [167]	Medical image segmentation (supervised)	Others	Multimodal (3D & 2D)
Zhou et al. [60]	SAM with multiple prompts using entropy	ISIC 2017 [72], CT2US [168], KITS23 [169], BraTS 2021 [68], Kvasir-SEG [119]	Medical image segmentation (supervised)	Foundation-model-based—prompt-induced/deterministic uncertainty	Multimodal (3D & 2D)
Lu et al. [61]	SAM with supervised fine-tuning and logit alignment through stochastic modeling	Left Atrium [132], Pancreas-CT [154]	Medical image segmentation (semi-supervised)	Bayesian uncertainty	3D MRI & CT
Shen et al. [62]	SAM with logits from multiple decoder	Total Segmentator [128]	Medical image segmentation (supervised)	Others	3D CT
Wei et al. [63]	SAM with uncertainty-guided sampling	Kvasir-Sessile [119], Multi-Class Organ BCV [170]	Medical image segmentation (supervised)	Deterministic methods	2D CT & colonoscopy
Zhang et al. [64]	SAM with prompt augmentation	StructSeg [171], FLARE22 [172]	Medical image segmentation (supervised)	Test-time augmentation	3D MRI & CT

## Data Availability

No new data were created or analyzed in this study. Data sharing is not applicable to this article.
